# Hobby engagement and hypertension risk: evidence from two large prospective cohorts

**DOI:** 10.1186/s12889-026-28750-x

**Published:** 2026-07-27

**Authors:** Jia-min Yan, Si-jing Liu, Min-zhe Zhang, Hong-jie Yu, Bang-xian Ding, Qi-qiang He

**Affiliations:** 1 Department of Laboratory Medicine, Wuhan Children’s Hospital (Wuhan Maternal and Child Healthcare Hospital), Tongji Medical College, Huazhong University of Science & Technology, Wuhan, P. R. China; 2 Children’s Medical Ward I, Wuhan Children’s Hospital (Wuhan Maternal and Child Healthcare Hospital), Tongji Medical College, Huazhong University of Science & Technology, Wuhan, P. R. China; 3 School of public health, Wuhan University, Wuhan, China; 4 Hubei Biomass-Resource Chemistry and Environmental Biotechnology Key Laboratory, Wuhan University, Wuhan, China

**Keywords:** Hobby engagement, Hypertension, Cohort study, CHARLS, HRS

## Abstract

**Objective:**

To investigate the association between hobby engagement and the risk of hypertension using data from the China Health and Retirement Longitudinal Study (CHARLS) and the Health and Retirement Study (HRS).

**Methods:**

This study included 7,112 participants aged ≥ 50 years from CHARLS and 4,937 participants aged ≥ 50 years from HRS. Hobby engagement was assessed at baseline and at follow-up, and longitudinal changes in hobby engagement were analyzed. The primary outcome was incident hypertension. Systolic blood pressure (SBP) and diastolic blood pressure (DBP) were also measured. Cox proportional hazards models were used to estimate hazard ratios (HRs) for incident hypertension, and changes in SBP and DBP with hobby engagement were examined using linear mixed-effects models. Kaplan-Meier curves and subgroup analyses were performed.

**Results:**

In baseline analyses, participants with hobby engagement had a significantly lower risk of developing hypertension compared to non-engaged participants in both CHARLS (HR = 0.84, 95% CI: 0.76,0.93) and HRS (HR = 0.86, 95% CI: 0.78,0.96) after full adjustment. In longitudinal analyses of hobby engagement patterns, persistent hobby engagement had the greatest protective effect in both cohorts compared with persistent hobby non-engagement in CHARLS (HR = 0.77, 95% CI: 0.68,0.88) and HRS (HR = 0.83, 95% CI: 0.73,0.95), respectively. Hobby engagement was associated with reductions in SBP (-0.52 mmHg per year) and DBP (-0.24 mmHg per year) over time in CHARLS. However, this effect was not observed in HRS.

**Conclusions:**

Hobby engagement is a significant protective factor against hypertension, with stronger benefits observed among individuals who maintained their hobby engagement over time. These findings support the integration of hobby-based interventions into public health strategies for preventing hypertension in middle-aged and older populations.

**Supplementary Information:**

The online version contains supplementary material available at https://doi.org/10.1186/s12889-026-28750-x.

## Introduction

Hypertension is one of the most significant threats to global public health by substantially increasing the risk of heart disease, stroke, chronic kidney disease and premature death, etc [1]. By 2025, the number of adults with hypertension is projected to exceed 1.5 billion worldwide [2]. As populations age globally, the burden of hypertension will continue to increase, making prevention strategies increasingly critical for public health [3, 4]. In particular, China and the United States are confronting considerable challenges to hypertension due to the large aging population.

Lifestyle factors play a critical role for the prevention of hypertension [5, 6]. Traditional behavioral measures such as weight management, physical activity, limiting alcohol intake, smoking cessation, and dietary improvements have demonstrated efficacy in blood pressure management. In recent years, increasing attention has turned toward psychosocial lifestyle behaviors that may contribute to cardiovascular health [7, 8]. Among these, hobby engagement has gained increasing attention as a potentially modifiable health behavior. Hobbies represent voluntary, personally meaningful activities that individuals pursue during their leisure time for pleasure and personal fulfillment [9]. Recent epidemiological research highlights the potential health benefits of hobby engagement. In a large multinational study covering 16 countries, it was found that engaging in hobbies was associated with a lower risk of depressive symptoms and better mental well-being among people aged 65 years and older [10]. Another comprehensive cross-national analysis including 19 countries found that engagement in hobbies was significantly associated with a lower risk of both all-cause and cause-specific mortality in adults aged 50 years and older [11]. However, the specific relationship between hobby engagement and hypertension remains poorly understood.

Therefore, the objective of this study was to investigate the association between hobby engagement and hypertension using data from two large cohorts. Specifically, we examined both baseline and longitudinal patterns of hobby engagement in relation to hypertension risk and the changes in blood pressure over time.

## Methods

### Study population

This study utilized data from the China Health and Retirement Longitudinal Study (CHARLS) and the Health and Retirement Study (HRS), two large-scale, nationally representative longitudinal cohorts that employed comparable methodologies and harmonized questionnaires, thereby enabling robust cross-national comparative analyses [12, 13]. Detailed descriptions of the CHARLS and HRS cohorts are provided in the Supplementary Materials. Both studies received ethical approval from their respective institutional review boards, and all participants provided written informed consent.

Participants aged 50 years and above were included in this study. The exclusion criteria were: (1) had memory-related disease and hypertension at baseline; (2) missed data on hobby engagement or key covariates; and (3) lost to follow-up. The final samples included 7,112 participants from CHARLS and 4,937 participants from HRS (Figure S1-S2).

### Exposure and outcome assessment

Hobby engagement was determined through self-reports. As the specific definitions varied between the CHARLS and HRS cohorts, a unified binary variable (Yes/No) was constructed for comparative analysis, which is consistent with recent literature on hobby engagement [10, 11]. The outcomes of hypertension, systolic blood pressure (SBP), and diastolic blood pressure (DBP) were determined through a combination of self-reported information and direct measurement. Detailed definitions of hobby engagement, hypertension, SBP and DBP for both CHARLS and HRS cohorts are provided in the Supplementary Materials (Methods S1-S2).

### Covariates

Covariates included a range of demographic, socioeconomic, lifestyle, and health-related factors known to be associated with hypertension risk. Demographic variables included age (continuous) and sex (male/female). Socioeconomic variables encompassed educational attainment (less than high school education vs. high school education or above) and marital status (married vs. single or other). Lifestyle factors included smoking status (current smoking vs. non-smoking), and drinking status (current drinking vs. non-drinking). Health-related variables included body mass index (BMI, kg/m^2^) status. All covariates were measured at baseline.

### Statistical analysis

Baseline characteristics of participants in both CHARLS and HRS cohorts were summarized according to hobby engagement status. Continuous variables were displayed as means ± standard deviations, and categorical variables were presented as frequencies ± percentages. Standardized mean differences (SMDs) were calculated to assess the magnitude of baseline imbalance between participants with and without hobby engagement, with an absolute SMD > 0.10 considered indicative of meaningful imbalance. Cox proportional hazards regression models were used to estimate hazard ratios (HRs) and 95% confidence intervals (CIs) for the association between baseline hobby engagement and incident hypertension. Three incrementally adjusted models were fitted as follows: Model 1 adjusted for age and sex; Model 2 additionally adjusted for education level, marital status, smoking status, and drinking status; Model 3 further adjusted for BMI status. The proportional hazards assumption was confirmed for all models using Schoenfeld residuals (all *P* > 0.05). To assess the robustness of the findings, several sensitivity analyses were performed: (1). excluding participants with chronic diseases (diabetes, heart disease, and stroke); (2). conducting multiple imputation for missing covariate data using the mice package to generate 20 imputed datasets (m = 20); (3). excluding participants who developed hypertension within the first two years of follow-up; (4). applying inverse probability of censoring weighting (IPCW); (5). conducting a complete-case analysis with additional adjustment for baseline SBP and DBP. Kaplan-Meier survival curves were constructed to visualize hypertension-free survival according to hobby engagement status. Subgroup analyses stratified by all covariates were conducted to explore potential effect modification.

To evaluate the impact of longitudinal changes on hobby engagement, we defined four distinct behavioral trajectories based on two consecutive exposure assessments during the follow-up period, according to established methodological frameworks [14]. Participants were categorized as follows: persistent hobby non-engagement, initiation of hobby engagement, cessation of hobby engagement, and persistent hobby-engagement (Table S1-S2). Those with persistent non-engagement were set as the reference group. Cox proportional hazards models were applied to examine the association between longitudinal hobby engagement patterns and incident hypertension.

Linear mixed-effects models were used to examine changes in SBP and DBP over time according to hobby engagement status. These models incorporated random intercepts and slopes to account for individual variations in blood pressure trajectories. Interaction terms between time and hobby engagement were included to test whether the change of blood pressure differed by engagement status.

All analyses were conducted separately for CHARLS and HRS cohorts using R (version 4.4.3). A two-sided P-value < 0.05 was considered statistically significant.

## Results

### Baseline characteristics

Table 1 presents baseline characteristics by hobby engagement status in CHARLS and HRS cohorts.

**Table 1:** Baseline characteristics of participants in CHARLS and HRS

Variables	CHARLS				HRS			
	**All participants** (*N* = 7112)	**Without hobby engagement** (*N* = 5496)	**With hobby engagement** (*N* = 1616)	*P*-value	**All participants** (*N* = 4937)	**Without hobby engagement** (*N* = 1924)	**With hobby engagement** (*N* = 3013)	*P*-value
Age (years)	61.37 (7.96)	61.55 (7.99)	60.78 (7.84)	< 0.001	66.76 (9.52)	68.16 (10.16)	65.86 (8.97)	< 0.001
Sex, n (%)				< 0.001				0.002
Male	3,585 (50.4)	2,611 (47.5)	974 (60.3)		2,041 (41.3)	743 (38.6)	1,298 (43.1)	
Female	3,527 (49.6)	2,885 (52.5)	642 (39.7)		2,896 (58.7)	1,181 (61.4)	1,715 (56.9)	
Education level, n (%)				< 0.001				< 0.001
Less than high school education	6,449 (90.7)	5,116 (93.1)	1,333 (82.5)		670 (13.6)	375 (19.5)	295 (9.8)	
High school education or above	663 (9.3)	380 (6.9)	283 (17.5)		4,267 (86.4)	1,549 (80.5)	2,718 (90.2)	
Marital status, n (%)				< 0.001				< 0.001
Married	6,148 (86.4)	4,698 (85.5)	1,450 (89.7)		3,346 (67.8)	1,196 (62.2)	2,150 (71.4)	
Single or other	964 (13.6)	798 (14.5)	166 (10.3)		1,591 (32.2)	728 (37.8)	863 (28.6)	
Smoking status, n (%)				< 0.001				0.062
Non-smoking	4,698 (66.1)	3,751 (68.2)	947 (58.6)		4,298 (87.1)	1,653 (85.9)	2,645 (87.8)	
Current smoking	2,414 (33.9)	1,745 (31.8)	669 (41.4)		639 (12.9)	271 (14.1)	368 (12.2)	
Drinking status, n (%)				< 0.001				< 0.001
Non-drinking	4,640 (65.2)	3,702 (67.4)	938 (58.0)		2,072 (42.0)	915 (47.6)	1,157 (38.4)	
Current drinking	2,472 (34.8)	1,794 (32.6)	678 (42.0)		2,865 (58.0)	1,009 (52.4)	1,856 (61.6)	
BMI (kg/m^2^)	22.72 (3.61)	22.52 (3.53)	23.42 (3.76)	< 0.001	26.87 (5.16)	26.96 (5.55)	26.81 (4.90)	0.319

The study included 7,112 participants from CHARLS (mean age 61.37 years, 50.4% male) and 4,937 participants from HRS (mean age 66.76 years, 41.3% male). In CHARLS, 1,616 (22.7%) participants reported hobby engagement at baseline, compared to 3,013 (61.0%) in HRS. In CHARLS, participants with hobby engagement were slightly younger (60.78 vs. 61.55 years), more likely to be male (60.3% vs. 47.5%) and married (89.7% vs. 85.5%), had higher education levels (17.5% vs. 6.9% high school education or above), and had higher BMI (23.42 vs. 22.52 kg/m^2^) than their counterparts. While in HRS, participants with hobby engagement were generally younger (65.88 vs. 68.16 years), more likely to be female (56.9% vs. 43.1%) and married (71.4% vs. 62.2%), and had higher education levels (90.2% vs. 80.5% high school education or above). Standardized mean differences showed several baseline differences between participants with and without hobby engagement in both cohorts (Table S3). In CHARLS, the largest imbalances were observed for education level (SMD = 0.33), sex (SMD = 0.26), smoking status (SMD = 0.20), overweight/obesity (SMD = 0.20), and drinking status (SMD = 0.19). In HRS, the largest imbalances were observed for education level (SMD = 0.28), age (SMD = 0.24), marital status (SMD = 0.20), and drinking status (SMD = 0.19).

### Association between baseline hobby engagement and incident hypertension

**Fig. 1:**
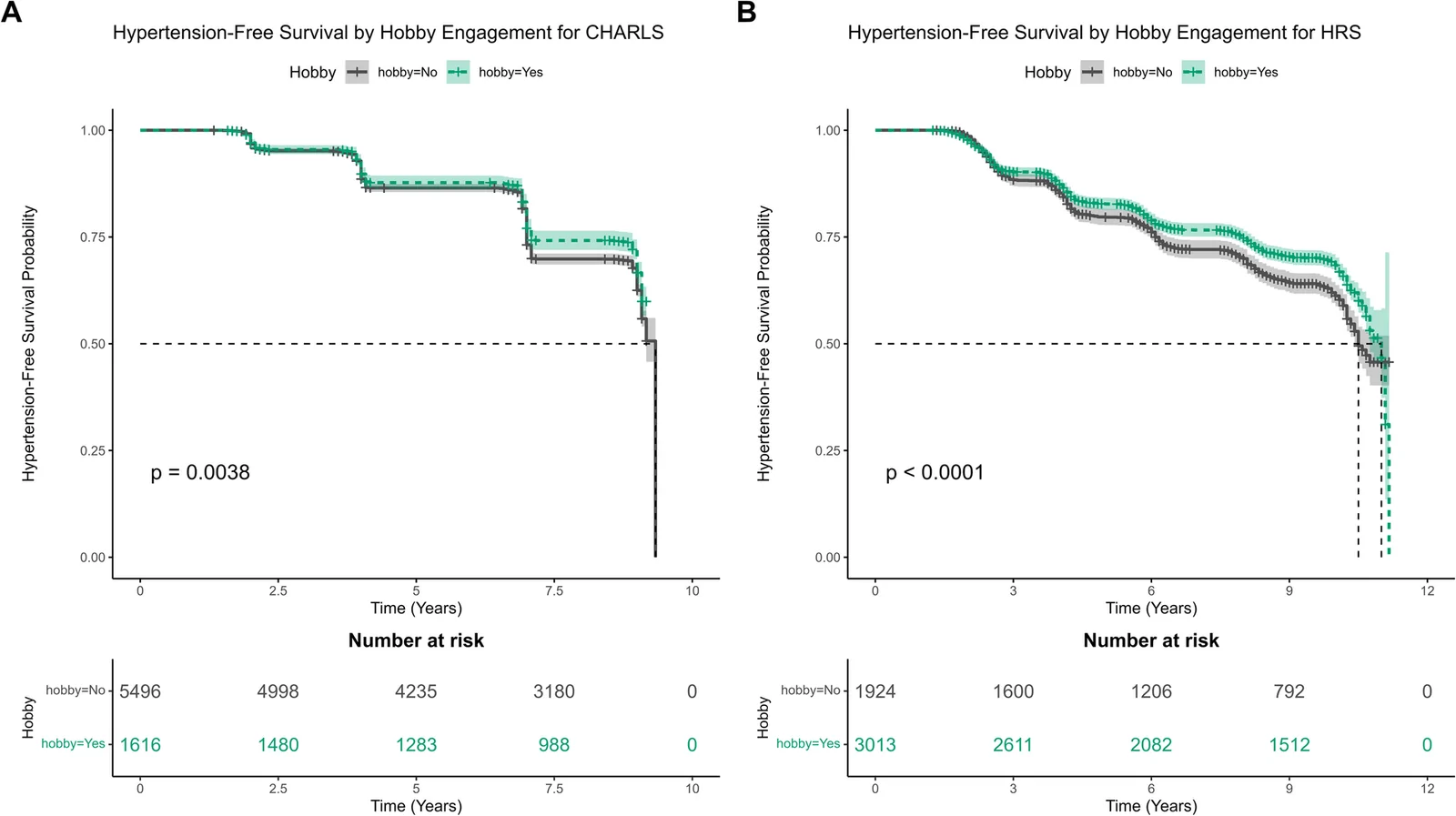
Kaplan-Meier Curves of hypertension-free survival probability by hobby engagement in the CHARLS and HRS Cohorts: **A** CHARLS; **B** HRS

Table 2 shows the association between baseline hobby engagement and incident hypertension. Over a follow-up period of 9.33 years in CHARLS and 11.17 years in HRS, participants contributed 51,975.0 and 36,568.3 person-years of follow-up, respectively. During the follow-up, 2,351 (33.1%) participants in CHARLS and 1,585 (32.1%) participants in HRS developed hypertension, respectively. In both cohorts, the incidence rate of hypertension was lower among participants with hobby engagement than among those without hobby engagement. Specifically, the incidence rates were 41.0 versus 46.5 per 1,000 person-years in CHARLS and 39.9 versus 49.2 per 1,000 person-years in HRS (Table S4). In fully adjusted models (Model 3), participants with hobby engagement exhibited a 16% lower risk for hypertension in CHARLS (HR = 0.84, 95% CI: 0.76,0.93, *P* < 0.001) and a 14% lower risk for hypertension in HRS (HR = 0.86, 95% CI: 0.78,0.96, *P* = 0.005), compared to those without hobby engagement. Sensitivity analyses yielded results consistent with the primary findings after excluding participants with baseline diabetes, heart disease, and stroke (Table S5), using multiple imputation (Table S6), excluding incident hypertension within the first two years, applying IPCW, and additionally adjusting for baseline SBP/DBP in complete cases (Tables S7-S9). Figure 1 displays Kaplan-Meier survival curves illustrating hypertension-free survival probability stratified by hobby engagement status. Consistently higher survival probabilities were observed among hobby-engaged participants throughout the follow-up period in both cohorts.

**Table 2:** Associations between hobby engagement and the risk of hypertension in CHARLS and HRS

Cohort	Hobby engagement	Model 1 h (95% CI), *P*-value	Model 2 h (95% CI), *P*-value	Model 3 h (95% CI), *P*-value
CHARLS				
	No	1.00 (Reference)	1.00 (Reference)	1.00 (Reference)
	Yes	**0.88 (0.79**,**0.97)**,** 0.010**	**0.89 (0.81**,**0.99)**,** 0.026**	**0.84 (0.76**,**0.93)**,** < 0.001**
HRS				
	No	1.00 (Reference)	1.00 (Reference)	1.00 (Reference)
	Yes	**0.82 (0.74**,**0.91)**,** < 0.001**	**0.86 (0.78**,**0.95)**,** 0.004**	**0.86 (0.78**,**0.96)**,** 0.005**

Hazard ratios (HRs) and 95% confidence intervals (CIs) were estimated using Cox proportional hazards models. Model 1 was adjusted for age and sex; Model 2 was further adjusted for education level, marital status, smoking status, and drinking status; Model 3 was additionally adjusted for overweight/obesity

Bold values indicate statistically significant results at *P* < 0.05

### Longitudinal patterns of hobby engagement and hypertension risk

The distribution of hobby engagement change patterns differed between the two cohorts (Table S2). Persistent non-engagement was the most prevalent pattern in CHARLS (4,245 participants, 66.7%), whereas persistent engagement was the most prevalent pattern in HRS (1,630 participants, 44.2%). Table 3 presents the association between longitudinal patterns of hobby engagement and hypertension risk. Compared to persistent non-engagement, persistent hobby engagement conferred a stronger protective effect in both cohorts. In the fully adjusted model, persistent engagement was associated with HRs of 0.77 (95% CI: 0.68,0.88, *P* < 0.001) in CHARLS and 0.83 (95% CI: 0.73,0.95, *P* = 0.007) in HRS, representing 23% and 17% risk reductions for hypertension, respectively.

**Table 3:** Associations between changes in hobby engagement and the risk of hypertension in CHARLS and HRS

Cohort	Hobby engagement patterns	Model 1 h (95% CI), *P*-value	Model 2 h (95% CI), *P*-value	Model 3 h (95% CI), *P*-value
CHARLS				
	Persistent non-engagement	1.00 (Reference)	1.00 (Reference)	1.00 (Reference)
	Initiation of engagement	0.94 (0.82,1.08), 0.377	0.95 (0.82,1.09), 0.445	0.89 (0.77,1.02), 0.102
	Cessation of engagement	0.91 (0.77,1.06), 0.226	0.92 (0.78,1.08), 0.288	0.86 (0.73,1.02), 0.076
	Persistent engagement	**0.82 (0.72**,**0.94)**,** 0.003**	**0.84 (0.74**,**0.95)**,** 0.007**	**0.77 (0.68**,**0.88)**,** < 0.001**
HRS				
	Persistent non-engagement	1.00 (Reference)	1.00 (Reference)	1.00 (Reference)
	Initiation of engagement	0.97 (0.79,1.19), 0.766	0.99 (0.80,1.21), 0.898	0.99 (0.80,1.21), 0.896
	Cessation of engagement	**0.83 (0.70**,**0.98)**,** 0.024**	**0.84 (0.71**,**0.99)**,** 0.041**	**0.84 (0.72**,**1.00)**,** 0.044**
	Persistent engagement	**0.78 (0.68**,**0.89)**,** < 0.001**	**0.83 (0.73**,**0.95)**,** 0.007**	**0.83 (0.73**,**0.95)**,** 0.007**

Hazard ratios (HRs) and 95% confidence intervals (CIs) were estimated using Cox proportional hazards models. Model 1 was adjusted for age and sex; Model 2 was further adjusted for education level, marital status, smoking status, and drinking status; Model 3 was additionally adjusted for overweight/obesity

Bold values indicate statistically significant results at *P* < 0.05

### Hobby engagement and blood pressure changes over time

Table 4 shows the association between hobby engagement and longitudinal changes in blood pressure. In CHARLS, hobby engagement significantly decreased blood pressure over time. In fully adjusted linear mixed-effects models, participants with hobby engagement had a significant reduction in SBP by 0.52 mmHg/year (95% CI: -0.90,-0.15, *P* = 0.006), and in DBP by 0.24 mmHg/year (95% CI: -0.46,-0.02, *P* = 0.031), compared with individuals without hobby engagement. In contrast, no statistically significant interaction effects were observed in the HRS cohort.

**Table 4:** Association between hobby engagement and changes in systolic and diastolic blood pressure over time

Cohort	Outcome	Term	Model 1 β (95% CI), *P*-value	Model 2 β (95% CI), *P*-value	Model 3 β (95% CI), *P*-value
CHARLS					
	SBP	Time (per year)	**0.28 (0.13**,**0.43)**,** < 0.001**	**0.28 (0.13**,**0.43)**,** < 0.001**	**0.28 (0.13**,**0.43)**,** < 0.001**
		Hobby engagement (Yes vs. No)	-0.79 (-2.16,0.58), 0.259	-0.95 (-2.33,0.44), 0.181	**-1.79** **(-3.17**,**-0.42)**,** 0.011**
		Time × Hobby engagement interaction	**-0.53 (-0.90**,** -0.16)**,** 0.005**	**-0.53 (-0.90**,** -0.16)**,** 0.005**	**-0.52 (-0.90**,** -0.15)**,** 0.006**
	DBP	Time (per year)	0.04 (-0.04,0.13), 0.328	0.05 (-0.04,0.14), 0.316	0.05 (-0.04,0.13), 0.321
		Hobby engagement (Yes vs. No)	-0.12 (-0.94,0.70), 0.772	-0.18 (-1.01,0.65), 0.669	-0.73 (-1.55,0.09) 0.081
		Time × Hobby engagement interaction	**-0.25** **(-0.47**,**-0.02)**,** 0.030**	**-0.25** **(-0.47**,**-0.02)**,** 0.030**	**-0.24** **(-0.46**,**-0.02)**,** 0.031**
HRS					
	SBP	Time (per year)	-0.07 (-0.23,0.09), 0.422	-0.06 (-0.22,0.10), 0.445	-0.06 (-0.22,0.10), 0.440
		Hobby engagement (Yes vs. No)	-1.19 (-2.5, 0.12), 0.076	-0.50 (-1.83, 0.82), 0.456	-0.48 (-1.80,0.84), 0.474
		Time × Hobby engagement interaction	0.02 (-0.18,0.23), 0.831	0.02 (-0.18,0.22), 0.842	0.02 (-0.18,0.22), 0.852
	DBP	Time (per year)	-0.41 (-0.51,-0.32), < 0.001	-0.41 (-0.51,-0.32), < 0.001	-0.41 (-0.51,-0.32), < 0.001
		Hobby engagement (Yes vs. No)	-0.36 (-1.16,0.44), 0.377	-0.17 (-0.98,0.63), 0.674	-0.15 (-0.95, 0.64), 0.705
		Time × Hobby engagement interaction	-0.06 (-0.18,0.06), 0.347	-0.06 (-0.18,0.06), 0.340	-0.06 (-0.18,0.06), 0.330

Results were estimated using linear mixed-effects models. Model 1 was adjusted for age and sex; Model 2 was further adjusted for education level, marital status, smoking status, and drinking status; Model 3 was additionally adjusted for overweight/obesity. A random intercept and random slope for time were included to account for within-individual and between-individual variations in blood pressure trajectories for all models

Bold values indicate statistically significant results at *P* < 0.05

### Subgroup analyses

**Fig. 2:**
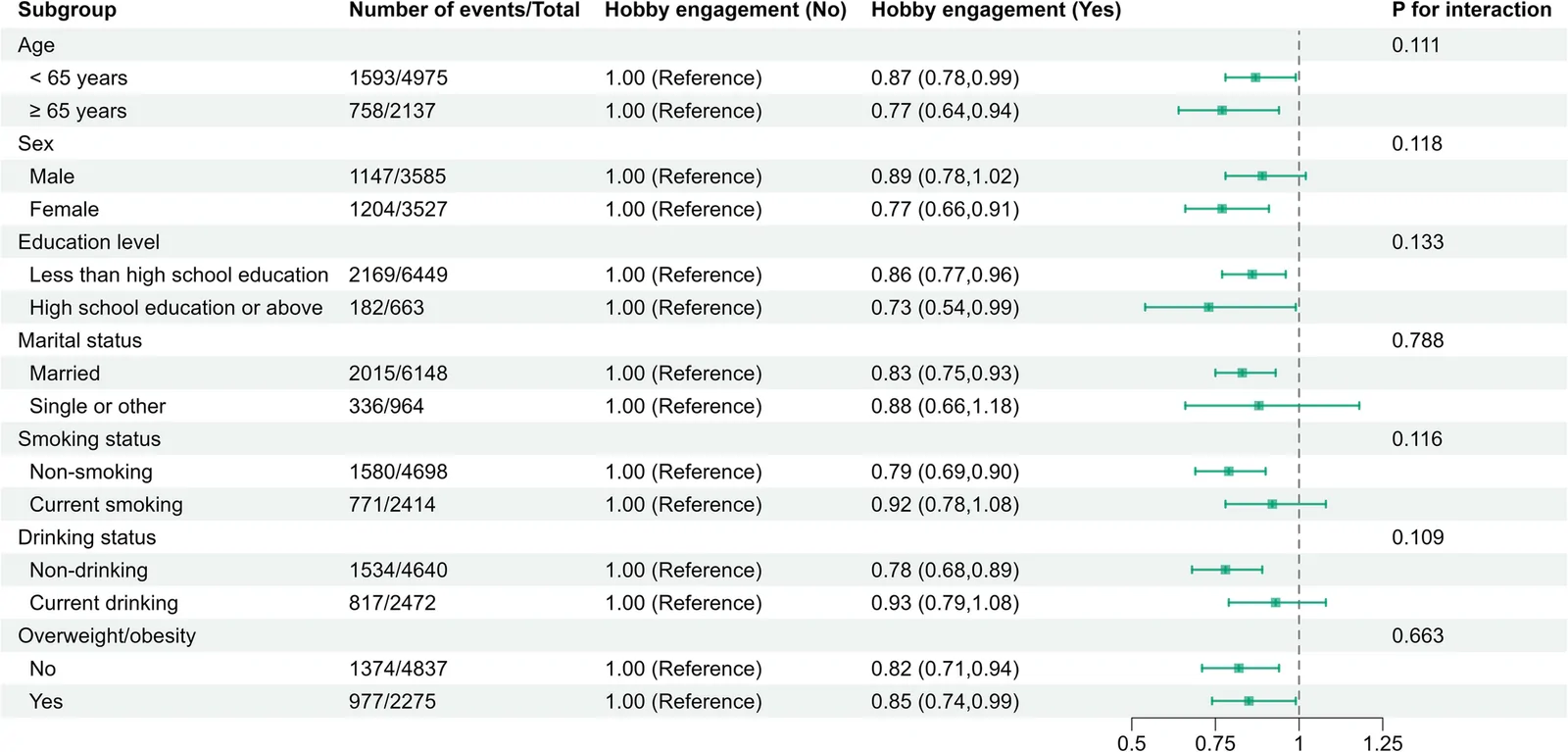
Subgroup analysis of the association between hobby engagement and hypertension in CHARLS

**Fig. 3:**
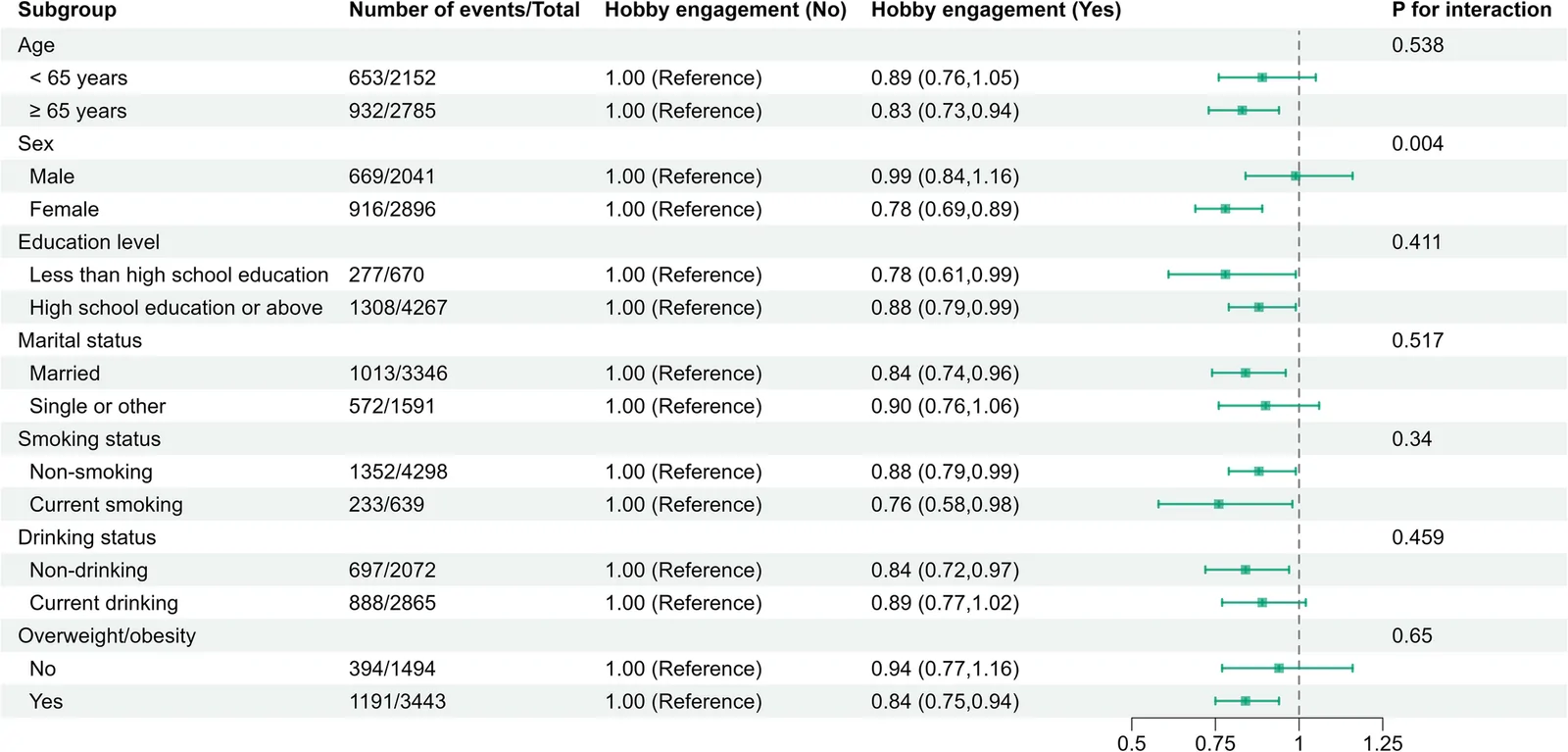
Subgroup analysis of the association between hobby engagement and hypertension in HRS

Figures 2 and 3 illustrate the results of subgroup analyses for the CHARLS and HRS cohorts, respectively. In CHARLS, no significant interaction was found between hobby engagement and incident hypertension across all subgroups. In contrast, a significant interaction by sex in HRS was identified (P for interaction = 0.004). Specifically, hobby engagement was associated with a reduced risk of hypertension among females (HR = 0.78, 95% CI: 0.69,0.89), but not among males (HR = 0.99, 95% CI: 0.84,1.16).

## Discussion

In this large-scale cohort study involving Chinese and American adults aged 50 years and older, hobby engagement was significantly associated with a reduced risk of developing hypertension. Our findings reveal consistent protective effects of hobby engagement across two culturally distinct populations, with baseline hobby engagement reducing hypertension risk by 16% in the Chinese cohort (CHARLS) and 14% in the American cohort (HRS). Notably, persistent hobby engagement over time demonstrated even stronger protective effects, with a decreased hypertension risk of 23% in CHARLS and 17% in HRS, respectively. Furthermore, in the CHARLS cohort, hobby engagement was associated with significant blood pressure reduction over time, with decreased SBP by 0.52 mmHg/year and decreased DBP by 0.24 mmHg/year, respectively. These findings show that hobby engagement was an effective lifestyle factor for hypertension prevention with potential implications for public health policy.

Our findings are in line with previous studies reporting beneficial effects of hobby engagement. For instance, a cohort study of 56,381 adults aged 40–59 years demonstrated that hobby engagement was associated with a reduced risk of cardiovascular disease (CVD) [15]. Specifically, participants with one hobby had a 10% lower risk of CVD, while those with multiple hobbies exhibited a 20% lower risk. Another cohort study including 22,377 individuals found that hobby engagement was associated with a lower risk of disabling dementia [16], while a 5-year cohort study of 567 older adults found that hobby non-engagement was independently associated with cognitive decline [17]. Our study builds upon this evidence base by linking hobby engagement to hypertension prevention.

The magnitude of the protective effects of hobby engagement on hypertension in this study is clinically meaningful when compared with other lifestyle factors. For example, a previous meta-analysis demonstrated that DASH diet interventions could reduce hypertension risk by 19% [18]. Similarly, a meta-analysis of 136,846 adults reported that high levels of leisure-time physical activity were associated with a 19% reduction in hypertension risk, while moderate levels were associated with an 11% reduction [19]. Our findings suggest that hobby engagement confers significant protective benefits (14–16% risk reduction) that are remarkably consistent with this established spectrum of lifestyle interventions, potentially representing an additional, more accessible strategy for individuals who may face barriers to traditional lifestyle modifications. Importantly, unlike some interventions that require significant behavioral change or medical supervision, hobby engagement offers the unique advantage of being inherently enjoyable and self-sustaining, which may enhance long-term adherence and population-level impact.

Several biological and psychosocial mechanisms may underlie the observed protective effects of hobby engagement against hypertension. First, hobby engagement offers a valuable way to relieve the heightened stress and mental exhaustion in today’s fast-paced society. Chronic stress activates the hypothalamic-pituitary-adrenal axis and sympathetic nervous system, leading to sustained elevation of cortisol, catecholamines, and other stress hormones that directly contribute to increased blood pressure through vasoconstriction and sodium retention [20, 21]. By offering a source of enjoyment and relaxation, hobby engagement may help attenuate these physiological stress responses and support vascular health. Second, hobby engagement usually incorporates elements of physical activity. Regular participation in moderate-intensity activities such as gardening has been shown to lower blood pressure [22]. The cumulative effect of regular, moderate physical activity through hobby engagement may contribute to improved blood pressure through enhanced insulin sensitivity, reduced arterial stiffness, and improved autonomic balance [23, 24]. Third, social engagement is an integral component of many hobby activities. Social isolation and loneliness have been identified as independent risk factors for hypertension [25–27]. Hobby engagement often involves social interaction, potentially mitigating this risk through enhanced social support networks and reduced perceived social isolation. Furthermore, participation in hobby-related clubs, workshops, or community events can facilitate exposure to health-related information and behavioral guidance, potentially contributing additional protective effects against hypertension development [28, 29].

The consistent protective effects of hobby engagement on hypertension across the two culturally distinct cohorts strengthen the external validity of our findings. However, there are some notable differences between the CHARLS and HRS cohorts. The positive effect of hobby engagement on blood pressure changes over time was observed only in the CHARLS cohort. Although the estimated reductions in SBP and DBP were modest at the individual level, even small reductions in population blood pressure have been associated with meaningful decreases in cardiovascular morbidity and mortality. Therefore, if sustained over time, the observed differences may have important implications for population health. The cohort-specific pattern, with SBP and DBP reductions observed only in CHARLS, may reflect the different types of hobbies, the social context of hobby engagement, as well as different healthcare systems and lifestyle habits between China and the United States. For example, common hobbies among older Chinese adults often include communal and physically active pursuits such as square dancing, tai chi and playing mahjong, which integrate moderate exercise with strong social bonds in community settings, potentially amplifying the cardiovascular benefits [30]. In contrast, HRS assessed hobby engagement using a broader hobby/project question and a rotating half-sample design, meaning that hobby-related information was not measured in the same way at every wave for all participants. This may have increased exposure measurement error or reduced the precision of longitudinal estimates, thereby attenuating associations with SBP and DBP changes. Additionally, differences in healthcare access, such as China’s universal coverage with a focus on preventive community health versus the US’s more fragmented system may further reinforce the impact of hobby engagement [31, 32]. The subgroup analyses indicated that the protective effect of hobby engagement was more pronounced among females. Women may have greater stress-reduction benefits from hobby engagement, as they typically exhibit more effective utilization of social support networks and emotion-focused coping strategies compared to men [33, 34]. Moreover, epidemiological evidence indicates that women could gain significantly greater survival and cardiovascular benefits from similar levels of leisure-time physical activity. In a US cohort study including more than 400,000 participants, women experienced a 24% reduction in cardiovascular and all-cause mortality with moderate-to-vigorous activity, compared to reductions of 14% and 15% in men, respectively [35]. These may explain the gender difference in the protective effects of hobby engagements. Although a significant interaction by sex was observed in HRS, this finding should be interpreted cautiously because multiple subgroup analyses may increase the probability of chance findings. Further studies are needed to confirm whether the association between hobby engagement and hypertension differs by sex.

Our study has several strengths. The two large nationally representative cohorts enhance the generalizability of the findings across diverse populations. In addition, the longitudinal design, incorporating multiple waves of data collection and follow-up over a decade, allowed for robust analyses of both baseline associations and dynamic changes in hobby engagement patterns. However, some limitations should be considered when interpreting these findings. First, the prevalence of hobby engagement was substantially lower in CHARLS than in HRS, which may partly reflect differences in the measurement of hobby engagement across the two cohorts. In CHARLS, hobby engagement was derived from participation in a limited set of social, cultural, community, volunteer, and educational activities, whereas HRS assessed the frequency of working on a hobby or project. Although a harmonized binary variable was constructed to improve comparability between cohorts, differences in the underlying measurements may have contributed to the observed variation in prevalence and may have introduced some degree of exposure misclassification. Furthermore, hobby engagement was self-reported and dichotomized into a binary variable, which may have failed to capture the type, diversity, intensity, or duration of different hobby activities. As a result, we were unable to examine potential dose-response relationships between hobby engagement and hypertension risk, and it is possible that different levels or frequencies of engagement may confer varying degrees of benefit. Future studies examining specific types of hobbies and the optimal “dose” of hobby engagement would inform more nuanced insights for clinical recommendations. Second, although we adjusted for multiple covariates, residual confounding from unmeasured factors (e.g. family history of hypertension, dietary patterns, psychosocial stress) cannot be entirely ruled out. Third, we cannot establish causality despite the longitudinal design, as reverse causation remains possible given that individuals with subclinical or emerging health conditions might be less likely to engage in hobbies.

## Conclusion

In summary, our findings indicate that hobby engagement may serve as an effective behavioral strategy for the prevention of hypertension among middle-aged and older adults in both China and the United States. Given the large global aging population and the increasing burden of hypertension, hobby engagement should be recommended as a potentially accessible, and cost-efficient approach to mitigate hypertension risk.

## Supplementary Information

Supplementary Material 1.

Supplementary Material 2.

Supplementary Material 3.

## Data Availability

The data used in this study are publicly available. The CHARLS and HRS data can be accessed upon application at (http://charls.pku.edu.cn/en), and (https://hrs.isr.umich.edu/data-products), respectively.
