# Supplementary material for: Hobby engagement and hypertension risk: evidence from two large prospective cohorts
Source: BMC Public Health. 2026 Jul 27;26:2727. doi: 10.1186/s12889-026-28750-x (PMC13629103; doi:10.1186/s12889-026-28750-x)
Supplement: Supplementary file 1 [file 12889_2026_28750_MOESM1_ESM.docx]

**Supplementary file to “Hobby engagement and hypertension Risk: Evidence from two large prospective cohorts”**

Supplementary Methods S1: Study population.

Supplementary Methods S2: Exposure and outcome assessment.

Figure S1: Flowchart of participant selection in CHARLS.

Figure S2: Flowchart of participant selection in HRS.

Table S1: Timeline of survey waves used to determine changes in hobby engagement status.

Table S2: Frequencies of hobby engagement change patterns in CHARLS and HRS.

Table S3: Standardized mean differences for baseline characteristics by hobby engagement.

Table S4: Person-years and incidence rates according to baseline hobby engagement.

Table S5: Associations between hobby engagement and the risk of hypertension in CHARLS and HRS after excluding participants with chronic diseases (diabetes, heart disease, and stroke) at baseline.

Table S6: Associations between hobby engagement and the risk of hypertension in CHARLS and HRS after multiple imputation.

Table S7: Associations between hobby engagement and the risk of hypertension in CHARLS and HRS after excluding incident hypertension within the first two years.

Table S8: Associations between hobby engagement and the risk of hypertension in CHARLS and HRS using inverse probability of censoring weighting (IPCW).

Table S9: Associations between hobby engagement and the risk of hypertension in CHARLS and HRS additionally adjusting for baseline SBP and DBP.

**Supplementary Methods S1:** **Study population**

*The China Health and Retirement Longitudinal Study (CHARLS)*

CHARLS is a nationally representative longitudinal survey of Chinese residents aged 45 years and older, designed to examine health, retirement, and aging patterns in China. The baseline survey was conducted in 2011-2012 (Wave 1), with subsequent follow-ups in 2013-2014 (Wave 2), 2015-2016 (Wave 3), 2018 (Wave 4), and 2020 (Wave 5). The study employed a multi-stage probability sampling strategy, covering 150 counties/districts and 450 villages/urban communities across 28 provinces, representing approximately 95% of the Chinese population.

*The Health and Retirement Study (HRS)*

HRS is a longitudinal study of community-dwelling adults aged 50 years and older in the United States, initiated in 1992 and conducted biennially. The survey used a multistage area probability sample, with oversampling of Black and Hispanic individuals to ensure representativeness. For the present analysis, we utilized data from Wave 9 (2008-2009) through Wave 14 (2018-2019), as consistent measurement of hobby engagement began in Wave 9. This timeframe was selected to align with the CHARLS follow-up period and ensure comparability across cohorts. In the HRS, hobby engagement was collected using a rotating half-sample design. Respondents were randomly assigned to one of two subsamples, with each subsample completing the hobby engagement questionnaire every four years. Specifically, one half-sample was first interviewed about hobby engagement in Wave 9 and subsequently followed up in Wave 11 and Wave 13. The other half-sample was first interviewed in Wave 10 and followed up in Wave 12 and Wave 14. Following the approach used in previous research[1, 2], we combined the two rotating half-samples into a unified analytic dataset.

**Supplementary Methods S2: Exposure and Outcome Assessment**

*Definition of hobby engagement in CHARLS and HRS*

In CHARLS, participants were asked if they had engaged in a list of activities in the last month (1. Played Ma-jong, played chess, played cards, or went to community club. 2. Went to a sport, social, or other kind of club. 3. Took part in a community-related organization. 4. Done voluntary or charity work. 5. Attended an education or training course. 6. None of these.). A binary variable was constructed, with “1” indicating participation in any of these activities and “0” indicating none.

In HRS, hobby engagement was assessed by asking participants how often they worked on a hobby or project. A binary variable was constructed, with “1” indicating any frequency of participation (ranging from daily to at least once a month) and “0” for no participation in the last month or never.

| Cohort | Question text | Definition |
| --- | --- | --- |
| CHARLS | Have you done any of these activities in the last month? 1. Played Ma-jong, played chess, played cards, or went to community club. 2. Went to a sport, social, or other kind of club. 3. Took part in a community-related organization. 4. Done voluntary or charity work. 5. Attended an education or training course. 6. None of these. | 1= any of the activities; 0=none |
| HRS | How often do you work on a hobby or project? | 1=any frequencies (ranging from daily to at least once a month); 0=not in the last month/never |

*Definition of Hypertension, SBP and DBP in CHARLS and HRS*

SBP and DBP in CHARLS and HRS were measured following a standardized protocol by trained interviewers to ensure data reliability. Before measurement, participants were asked to sit down, relax and remain quiet for a period of time. Omro HEM-7200 monitor and Omron HEM-780 Intellisense Automated blood pressure monitor were used for CHARLS and HRS on the participant’s left arm and three repeated measurements were recorded with 45-second intervals between each reading. The final SBP and DBP values were recorded as the average of the second and third successful measurements taken avoid the potential “white coat effect”. Hypertension was defined as self-reported physician diagnosis or self-reported use of antihypertensive medications, or a measured SBP ≥140 mmHg or DBP ≥90 mmHg.

**Figure S1** Flowchart of participant selection in CHARLS.

**Figure S2** Flowchart of participant selection in HRS.

**Table S1** Timeline of survey waves used to determine changes in hobby engagement status.

| Cohort | Waves Used to Define Hobby Engagement Change Patterns | Waves Used for Hypertension Status Assessment |
| --- | --- | --- |
| **CHARLS** | Wave 1 (2011/2012) and Wave 2 (2013/2014) | Wave 3 (2015/2016), Wave 4 (2018), Wave 5 (2020) |
| **HRS** | Wave 9 (2008/2009) and Wave 11 (2012/2013) | Wave 12 (2014/2015), Wave 13 (2016/2017/2018), Wave 14 (2018/2019) |

**Table S2** Frequencies of hobby engagement change patterns in CHARLS and HRS.

| Cohort | Frequencies (%) | | | |
| --- | --- | --- | --- | --- |
|  | Persistent non-engagement | Initiation of engagement | Cessation of engagement | Persistent engagement |
| **CHARLS** | 4245 (66.7%) | 695 (10.9%) | 496 (7.8%) | 924 (14.5%) |
| **HRS** | 1032 (28.0%) | 332 (9.0%) | 697 (18.9%) | 1630 (44.2%) |

**Table S3** Standardized mean differences for baseline characteristics by hobby engagement.

| Cohort | Variable | SMD |
| --- | --- | --- |
| **CHARLS** | Age | 0.096 |
|  | Sex | 0.258 |
|  | Education level | 0.328 |
|  | Marital status | 0.129 |
|  | Smoking status | 0.201 |
|  | Drinking status | 0.193 |
|  | Overweight obesity | 0.200 |
| **HRS** | Age | 0.240 |
|  | Sex | 0.091 |
|  | Education level | 0.277 |
|  | Marital status | 0.196 |
|  | Smoking status | 0.055 |
|  | Drinking status | 0.186 |
|  | Overweight obesity | 0.007 |

**Table S4** Person-years and incidence rates according to baseline hobby engagement.

| Cohort | Hobby engagement | Participants | Events | Person-years | Incidence per 1,000 person-years |
| --- | --- | --- | --- | --- | --- |
| **CHARLS** | No | 5496 | 1859 | 39973.8 | 46.5 |
|  | Yes | 1616 | 492 | 12001.2 | 41.0 |
| **HRS** | No | 1924 | 670 | 13627.3 | 49.2 |
|  | Yes | 3013 | 915 | 22941.0 | 39.9 |

**Table S5** Associations between hobby engagement and the risk of hypertension in CHARLS and HRS after excluding participants with chronic diseases (diabetes, heart disease, and stroke) at baseline.

| Cohort | Hobby engagement | Model 1 HR (95% CI), P-value | Model 2 HR (95% CI), P-value | Model 3 HR (95% CI), P-value |
| --- | --- | --- | --- | --- |
| **CHARLS** |  |  |  |  |
|  | No | 1.00 (Reference) | 1.00 (Reference) | 1.00 (Reference) |
|  | Yes | **0.85 (0.76,0.95), 0.005** | **0.87 (0.77,0.97), 0.012** | **0.81 (0.72,0.91), <0.001** |
| **HRS** |  |  |  |  |
|  | No | 1.00 (Reference) | 1.00 (Reference) | 1.00 (Reference) |
|  | Yes | **0.80 (0.71,0.90), <0.001** | **0.84 (0.75,0.95), 0.004** | **0.84 (0.75,0.95), 0.005** |

Hazard ratios (HRs) and 95% confidence intervals (CIs) were estimated using Cox proportional hazards models. Model 1 was adjusted for age and sex; Model 2 was further adjusted for education level, marital status, smoking status, and drinking status; Model 3 was additionally adjusted for overweight/obesity.

**Table S6** Associations between hobby engagement and the risk of hypertension in CHARLS and HRS after multiple imputation.

| Cohort | Hobby engagement | Model 1 HR (95% CI), P-value | Model 2 HR (95% CI), P-value | Model 3 HR (95% CI), P-value |
| --- | --- | --- | --- | --- |
| **CHARLS** |  |  |  |  |
|  | No | 1.00 (Reference) | 1.00 (Reference) | 1.00 (Reference) |
|  | Yes | **0.87 (0.80,0.96), 0.004** | **0.89 (0.81,0.98), 0.015** | **0.84 (0.76,0.92), <0.001** |
| **HRS** |  |  |  |  |
|  | No | 1.00 (Reference) | 1.00 (Reference) | 1.00 (Reference) |
|  | Yes | **0.81 (0.74,0.90), <0.001** | **0.85 (0.77,0.94), 0.002** | **0.86 (0.77,0.95), 0.002** |

Hazard ratios (HRs) and 95% confidence intervals (CIs) were estimated using Cox proportional hazards models. Model 1 was adjusted for age and sex; Model 2 was further adjusted for education level, marital status, smoking status, and drinking status; Model 3 was additionally adjusted for overweight/obesity.

**Table S7** Associations between hobby engagement and the risk of hypertension in CHARLS and HRS after excluding incident hypertension within the first two years.

| Cohort | Hobby engagement | Model 1 HR (95% CI), P-value | Model 2 HR (95% CI), P-value | Model 3 HR (95% CI), P-value |
| --- | --- | --- | --- | --- |
| **CHARLS** |  |  |  |  |
|  | No | 1.00 (Reference) | 1.00 (Reference) | 1.00 (Reference) |
|  | Yes | **0.87 (0.79,0.97), 0.011** | **0.89 (0.80,0.99), 0.032** | **0.84 (0.75,0.93), 0.001** |
| **HRS** |  |  |  |  |
|  | No | 1.00 (Reference) | 1.00 (Reference) | 1.00 (Reference) |
|  | Yes | **0.79 (0.71,0.88), <0.001** | **0.83 (0.75,0.92), <0.001** | **0.83 (0.75,0.93), <0.001** |

Hazard ratios (HRs) and 95% confidence intervals (CIs) were estimated using Cox proportional hazards models. Model 1 was adjusted for age and sex; Model 2 was further adjusted for education level, marital status, smoking status, and drinking status; Model 3 was additionally adjusted for overweight/obesity.

**Table S8** Associations between hobby engagement and the risk of hypertension in CHARLS and HRS using inverse probability of censoring weighting (IPCW).

| Cohort | Hobby engagement | Model 1 HR (95% CI), P-value | Model 2 HR (95% CI), P-value | Model 3 HR (95% CI), P-value |
| --- | --- | --- | --- | --- |
| **CHARLS** |  |  |  |  |
|  | No | 1.00 (Reference) | 1.00 (Reference) | 1.00 (Reference) |
|  | Yes | **0.88 (0.79,0.97), 0.009** | **0.89 (0.80,0.99), 0.025** | **0.84 (0.75,0.93), <0.001** |
| **HRS** |  |  |  |  |
|  | No | 1.00 (Reference) | 1.00 (Reference) | 1.00 (Reference) |
|  | Yes | **0.82 (0.74,0.91), <0.001** | **0.86 (0.78,0.95), 0.004** | **0.86 (0.78,0.96), 0.005** |

Hazard ratios (HRs) and 95% confidence intervals (CIs) were estimated using inverse probability of censoring weighted Cox proportional hazards models with robust standard errors. Model 1 was adjusted for age and sex; Model 2 was further adjusted for education level, marital status, smoking status, and drinking status; Model 3 was additionally adjusted for overweight/obesity.

**Table S9** Associations between hobby engagement and the risk of hypertension in CHARLS and HRS additionally adjusting for baseline SBP and DBP.

| Cohort | Complete-case N | Events | HR (95% CI), P-value |
| --- | --- | --- | --- |
| **CHARLS** | 7063 | 2330 | **0.86 (0.77, 0.95), 0.003** |
| **HRS** | 2124 | 722 | **0.77 (0.66, 0.90), <0.001** |

Hazard ratios (HRs) and 95% confidence intervals (CIs) were estimated using Cox proportional hazards models. Model was adjusted for age, sex, education level, marital status, smoking status, and drinking status, overweight/obesity, baseline SBP and DBP.

**References**

1.	Gao Q, Bone JK, Bu F, Paul E, Sonke JK, Fancourt D: **Associations of Social, Cultural, and Community Engagement With Health Care Utilization in the US Health and Retirement Study**. *JAMA Netw Open* 2023, **6**(4):e236636.

2.	Guo Y, Yang F: **Hobby engagement and all-cause and cause-specific mortality risk among people aged 50 years and older in 19 countries**. *J Glob Health* 2025, **15**:04181.
